# Static Magnetic Field Attenuates Lipopolysaccharide-Induced Inflammation in Pulp Cells by Affecting Cell Membrane Stability

**DOI:** 10.1155/2015/492683

**Published:** 2015-03-26

**Authors:** Sung-Chih Hsieh, Jeng-Ting Tsao, Wei-Zhen Lew, Ya-Hui Chan, Lin-Wen Lee, Che-Tong Lin, Yung-Kai Huang, Haw-Ming Huang

**Affiliations:** ^1^School of Dentistry, College of Oral Medicine, Taipei Medical University, Taipei 11031, Taiwan; ^2^Department of Dentistry, Wan Fang Hospital, Taipei 11696, Taiwan; ^3^Division of Allergy and Immunology, Department of Internal Medicine, Cathay General Hospital, Taipei 10630, Taiwan; ^4^Department of Microbiology and Immunology, Taipei Medical University, Taipei 11031, Taiwan; ^5^School of Oral Hygiene, College of Oral Medicine, Taipei Medical University, 250 Wu-Hsing Street, Taipei 11031, Taiwan; ^6^Graduate Institute of Biomedical Materials and Tissue Engineering, College of Dental Medicine, Taipei Medical University, 250 Wu-Hsing Street, Taipei 11031, Taiwan

## Abstract

One of the causes of dental pulpitis is lipopolysaccharide- (LPS-) induced inflammatory response. Following pulp tissue inflammation, odontoblasts, dental pulp cells (DPCs), and dental pulp stem cells (DPSCs) will activate and repair damaged tissue to maintain homeostasis. However, when LPS infection is too serious, dental repair is impossible and disease may progress to irreversible pulpitis. Therefore, the aim of this study was to examine whether static magnetic field (SMF) can attenuate inflammatory response of dental pulp cells challenged with LPS. In methodology, dental pulp cells were isolated from extracted teeth. The population of DPSCs in the cultured DPCs was identified by phenotypes and multilineage differentiation. The effects of 0.4 T SMF on DPCs were observed through MTT assay and fluorescent anisotropy assay. Our results showed that the SMF exposure had no effect on surface markers or multilineage differentiation capability. However, SMF exposure increases cell viability by 15%. In addition, SMF increased cell membrane rigidity which is directly related to higher fluorescent anisotropy. In the LPS-challenged condition, DPCs treated with SMF demonstrated a higher tolerance to LPS-induced inflammatory response when compared to untreated controls. According to these results, we suggest that 0.4 T SMF attenuates LPS-induced inflammatory response to DPCs by changing cell membrane stability.

## 1. Introduction

Pulpitis is a disease caused by inflammation of dental pulp. When such inflammation occurs, pressure inside pulp cavity increases that cannot be dissipated by surrounding soft tissue because pulp is surrounded by hard tissue [1]. Since pulp contains blood vessels and nerves, pressure created by pulpitis causes pain and creates difficulties for blood and nutrient supply.

It is well known that pulp tissue is composed of immune cells, ectomesenchymal cells, fibroblasts, preodontoblasts, odontoblasts, and dental pulp stem cells (DPSCs) [2]. Of these cells, DPSCs exhibit multipotent differentiation ability; thus, tissue engineering study has gradually come to focus on DPSCs [3, 4]. In addition, DPSCs were reported to have excellent potential for dentin repair and tooth regeneration [5]. Given this potential use in tissue engineering, investigations into the treatment of bacterial induced pulpitis and tooth preservation are increasingly important for regenerative medicine. Until now, however, the only way to prevent pain is by removing the pulp via root canal treatment or tooth extraction. In this regard, several scholars have focused their aim on investigating the immunoresponse of DPSCs and dental pulp cells (DPCs) [5–9].

The gram-negative bacterial cell wall component lipopolysaccharide (LPS) is now well documented as an initiator of pulpitis. Among gram-negative bacteria,* Porphyromonas gingivalis* can be found in 48% of teeth with endodontic infection [10]. In addition, Botero et al. demonstrated that* Porphyromonas *endodontalis LPS induce cytokine expression in DPSCs and DPCs [7]. It is now known that the coreceptor of LPS formed by the Toll-like receptor 4 (TLR4) and CD14 is the binding site for signaling LPS-induced cytotoxicity [11, 12]. Even though DPSCs and DPCs express LPS receptors (TLRs) on their membrane surfaces [5, 7, 9], it is hard to bring medicines to the infected pulp tissue because these sites are surrounded by hard tissue. For the successful regeneration of pulp tissue in a root canal, neutralizing the adverse effects of residual LPS remains a challenge for scientists [5].

Static magnetic fields (SMFs) are physical stimulators that have anti-inflammatory effects on human macrophages and lymphocytes [13, 14] and on cytokine release by human peripheral blood mononuclear cells [15]. In an in vitro study, Lin et al. found that long-term SMF exposure inhibits LPS-induced cytotoxicity of fibroblasts [16]. Shen et al. also found that SMF attenuates lipopolysaccharide-induced neuroinflammatory response [17]. After an animal study, Lin's group showed for the first time that LPS-injected mice that had been preexposed to an SMF exhibited significantly better survival rates compared to unexposed control mice [18]. All these studies suggest that SMF has the potential to be an alternative stimulation source for controlling LPS-induced inflammatory response. Nevertheless, no study has yet investigated the anti-inflammatory effects of SMF on dental pulp cells. The aim of this study was to test whether or not SMF had attenuating effects on inflammatory response of LPS stimulated dental pulp cells.

## 2. Materials and Methods

### 2.1. DPCs Isolation and Culture

Human dental pulp was obtained from healthy wisdom teeth or orthodontically extracted premolars under the approval of the TMU-Joint Institutional Review Board. Freshly extracted teeth were immediately cleaned with Dulbecco's phosphate-buffered saline and sent to the lab for storage in a culture medium. The isolation method was modified from the outgrowth method discussed in previous studies [19–21]. The crown portions were separated using a sterile mortar and pestle after PBS irrigation. Pulp tissue was extirpated with forceps and sliced into small pieces with a scalpel. These small prices of minced pulp tissues were then cultured on 3.5 cm petri dishes using *α*-minimal essential medium (*α*-MEM; Gibco/Invitrogen, Grand Island, NY) supplemented with 15% FBS (Gibco/Invitrogen, Grand Island, NY), 100 *μ*M L-ascorbic acid 2-phosphate (Sigma-Aldrich, St. Louis, MO), and 1% antibiotic-antimycotic (Gibco/Invitrogen, Grand Island, NY) at 37°C in 5% CO_2_. Until reaching 70–80% confluence, DPCs were further cultured in new 10 cm petri dishes for further propagation.

### 2.2. SMF Equipment Setup and Exposure

**Figure 1:**
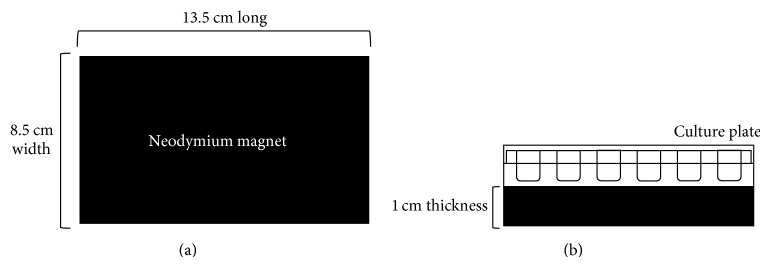
Schematic representation of the static magnetic field equipment setup. (a) Neodymium blocks 13.5 × 8.5 × 1 cm were used to provide a 0.4 T static magnetic field. (b) The 24-well culture plate was placed directly on the north pole (on the base) of the magnetic block.

A rectangular neodymium magnetic block 8.5 cm wide, 13.5 cm long, and 1 cm thick (Figure 1) with a 0.4 T flux density was used in our experiment to generate the SMF exposure environment. For the experimental group, DPCs were seeded on 24-well plates and placed on the north-pole surface of the neodymium magnet block for SMF stimulation. For sham groups, the cell culture dishes were placed on another similar but nonmagnetized neodymium block.

### 2.3. DPSC Identification

To identify the DPSCs population of the cultured DPCs, a set of surface markers were determined and differential stimulation was performed. Cell surface markers were labeled with corresponding antibodies and analyzed by flow cytometry. The DPCs numbering 1 × 10^5^ were cultured on 10 cm petri dishes and placed on the 0.4 T magnetic block for a period of 5 days. Then cells were collected and fixed with 75% ethanol at −20°C overnight. The fixed cells were incubated with the following fluorescent-conjugated antibodies in PBS at 4°C for 30 minutes: CD14 (AbD Serotec, NC, USA), CD34 (Santa Cruz Biotechnology, Santa Cruz, CA, USA), CD29 (Exbio, Praha, Czech Republic), CD73 (BD, Biosciences, Heidelberg, Germany), CD90, CD105 (Biolegend, San Diego, CA, USA), and CD146 (Santa Cruz). The cell suspension was then analyzed by flow cytometry (Guava EasyCyte Mini Base System, Guava Technologies, Millipore, Hayward, CA, USA) and raw data were analyzed by FlowJo software (TreeStar Inc., Ashland, USA).

The differentiation induction method was performed according to a previous published report [22]. Briefly, dental pulp cell solutions with a concentration of 2 × 10^4^ cells/mL were cultured in 24-well plates for further differential induction. The 24-well plates were placed on the magnetized block or sham block for the whole culturing period. At the same time, basal medium was changed to osteogenesis, adipogenesis, and chondrogenesis induction medium, respectively, and cultured for 1 month. During this induction period, the cultured cell medium was changed twice a week. For the osteogenesis stimulus, 1.8 mM KH_2_PO_4_ and 0.01 *μ*M dexamethasone were supplemented into the complete culture medium. For the adipogenesis stimulus, 10 *μ*g/mL insulin, 0.5 *μ*M hydrocortisone, 500 *μ*M IBMX, and 60 *μ*M indomethacin were supplemented into the culture medium. For the chondrogenesis stimulus, 0.1 *μ*M dexamethasone, 10 ng/mL TGF-*β*, and 1 mM sodium pyruvate were added to the serum-free complete medium. Control cells were cultured with basal medium on the magnetized or nonmagnetized neodymium magnet for the whole experimental period. At the end of the period, cells were fixed with 4% paraformaldehyde and stained with 2% Alizarin Red S, Oil Red O, and 1% Safranin O for calcium deposition, intracellular lipid droplet, or glycosaminoglycan observation, respectively. Stained cells were then washed with PBS several times and observed under an optical microscope (Nikon Eclipse TS100, Japan).

### 2.4. The Effect of 0.4 T SMF on DPC Proliferation

MTT assays were performed to determine the effect of 0.4 T SMF on DPC proliferation. Cell solutions with a concentration of 2 × 10^4^ cells/mL were seeded in two identical 24-well plates and placed on the surface of either the magnetized or nonmagnetized magnetic block for 5 days. Fifty *μ*L of tetrazolium salt (MTT) was added according to the supplier's instructions (MTT kit, Roche Applied Science, Mannheim, Germany) every 24 hours. After standing for 4 hours, formazan dye was solubilized by the addition of 500 *μ*L dimethyl sulfoxide (DMSO) and quantitated using a microplate reader (Model 2020, Anthos Labtec Instruments, Wals, Austria) at 570/690 nm. The optical density (OD) absorbance value was directly correlated to DPC number.

### 2.5. LPS Challenge to SMF-Exposed DPCs

To test the effect of the SMF on LPS-induced inflammatory response of DPCs, cells were starved in a serum-free medium for 12 hours. After being washed with PBS, cells were incubated with commercial* Pseudomonas aeruginosa *derived LPS (Sigma) at serial diluted concentrations ranging from 600 *μ*g/mL to 0 *μ*g/mL. After 12 hours, the MTT assay was performed to evaluate the viability of SMF-exposed and sham-exposed cells. Further, the cell morphology of SMF-exposed or sham-exposed LPS-challenged DPCs was observed with an optical microscope (Nikon Eclipse TS100, Japan).

### 2.6. Membrane Fluidity Measurement by Fluorescent Anisotropy

For the membrane fluidity test, 100 *μ*L cell solutions with a concentration of 5 × 10^4^ cells/mL were cultured in 96-well black plates for 24 hours. Then the cells were placed in the magnetic environments for an additional 8 hours. After discarding the culturing medium, 100 *μ*L of 1 *μ*M TMA-DPH or DPH was added to each well to label the cell membrane. Then cells were analyzed with a multilabel plate reader. Excitation and emission wavelengths were set at 355 nm and 430 nm, respectively. Fluorescent anisotropy was calculated using the following equation [23]:

(1) $$\begin{array}{c} r=\frac{\left(I_{||}-I_{⊥}\right)}{\left(I_{||}+2I_{⊥}\right)}, \end{array}$$

where *I*_||_ is fluorescence intensity measured through vertical excitation and vertical emission polarization filters and *I*_⊥_ is the analog measured through vertical excitation and horizontal emission polarization filters. Higher levels of fluorescent anisotropy indicate a decrease in dye mobility and increase in membrane structural order.

### 2.7. Statistical Analysis

The cell proliferation, cell viability, and cell membrane fluorescent anisotropy data were presented using descriptive statistics. Comparisons of means between SMF-exposed group and sham-exposed groups were performed using unpaired Student's *t*-test. The significance level was set at *P* < 0.05.

## 3. Results

**Figure 2:**
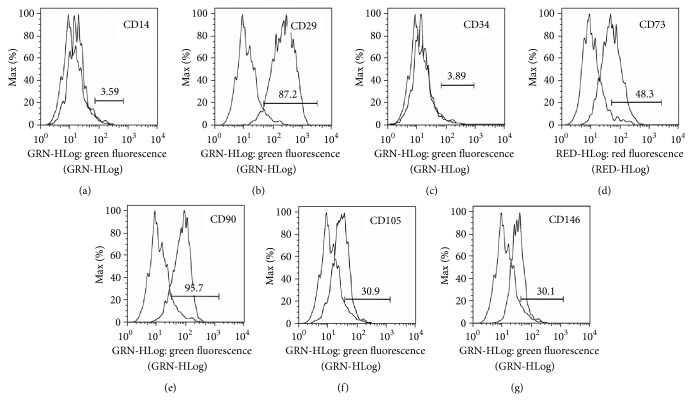
Flow cytometry histograms showed the DPSCs surface marker expressions after 0.4 T SMF exposure. Unstained control cells and cells stained with antibodies against the surface proteins were overlapped. Brackets indicate the positive cell populations in percent.

Cell surface markers were labeled with fluorescent-conjugated antibodies and analyzed by flow cytometry. The results demonstrated that the CD markers of SMF-exposed group were not different from previous published studies. It is positive for CD29, CD73, CD90, CD105, and CD146 and negative for CD14 and CD34 (Figure 2). There were high expression in CD29 (87.2%) and CD90 (95.7%) and moderate expression in CD73 (48.3%), CD105 (30.9%), and CD146 (30.1%), indicating that cells exhibit mesenchymal stem cell-like phenotypes even after prolonged culturing in SMF environment.

**Figure 3:**
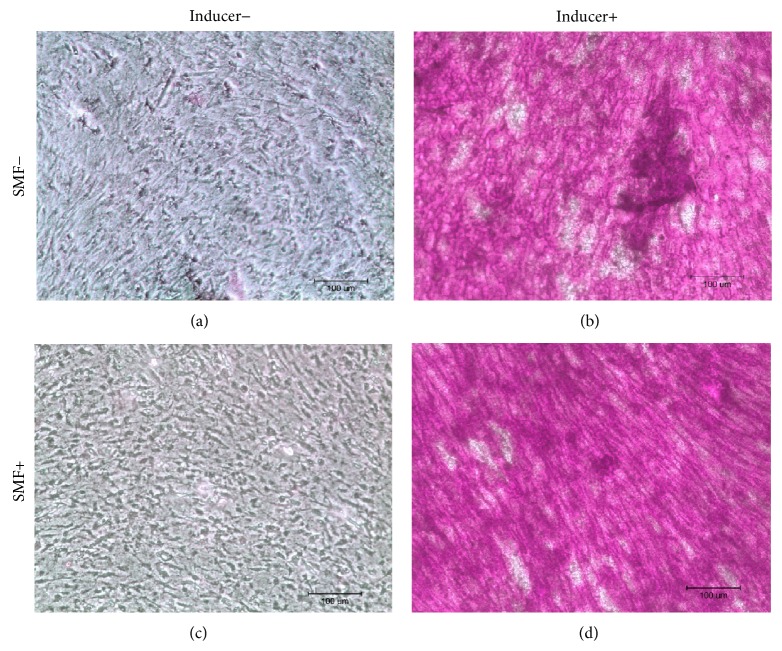
Results of Alizarin Red staining showed the calcified deposition in red. There were no significant differences between SMF-exposed and sham-exposed cells after osteogenesis induction. Neither of noninduction controls had calcified deposition present.

**Figure 4:**
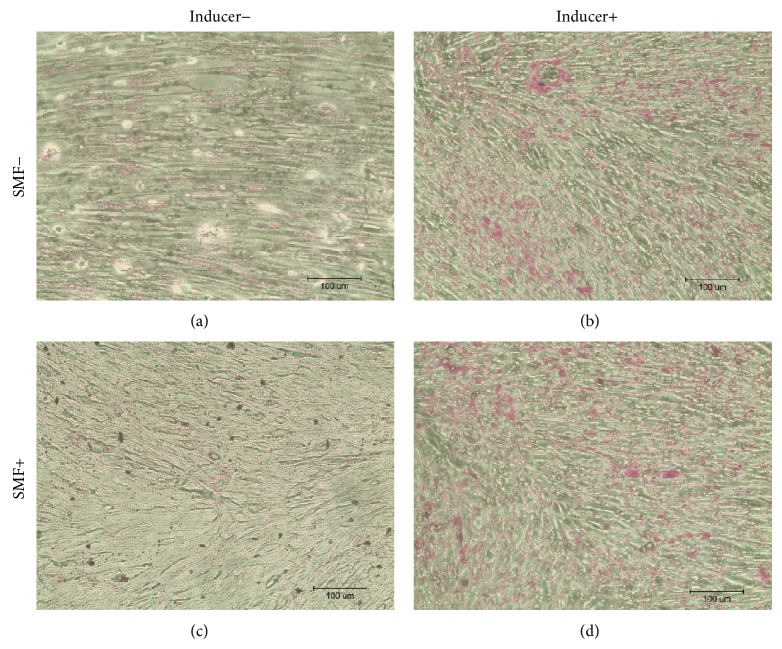
Oil Red O staining results showed the intracellular lipid droplets stained in red after adipogenesis induction. No significant differences could be observed between SMF-exposed and sham-exposed DPCs after adipogenesis induction.

**Figure 5:**
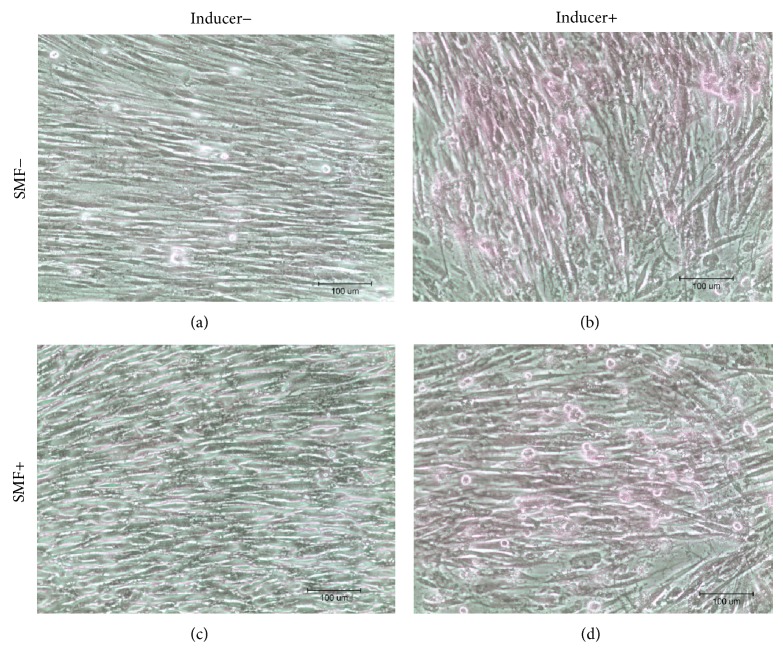
Safranin O staining results showed the glycosaminoglycan extracellular matrix around the cells in pink to red after chondrogenesis induction. No significant differences could be observed between SMF-exposed and sham-exposed DPCs after adipogenesis induction.

After culturing DPCs with the differentiation induction medium, the cells were stained with Alizarin Red, Oil Red O, and Safranin O. Observed under an optical microscope, sporadic calcified nodules in osteogenesis cells (Figure 3), intracellular lipid droplets in adipogenesis cells (Figure 4), and glycosaminoglycan matrix around the chondrogenesis cells (Figure 5) were found. The differentiation capability of SMF-exposed group showed no obvious superiority to the sham-exposed group, in neither osteogenesis, adipogenesis, nor chondrogenesis induction.

**Figure 6:**
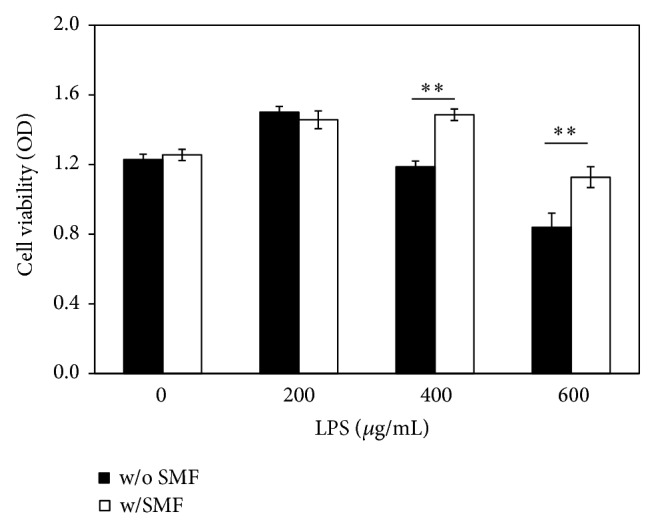
DPC cell grown after prolonged culturing in an SMF environment was enhanced. Cell viability of the SMF-exposed group was significantly higher than the sham-exposed group (*P* < 0.001) at day 3 and day 4.

**Figure 7:**
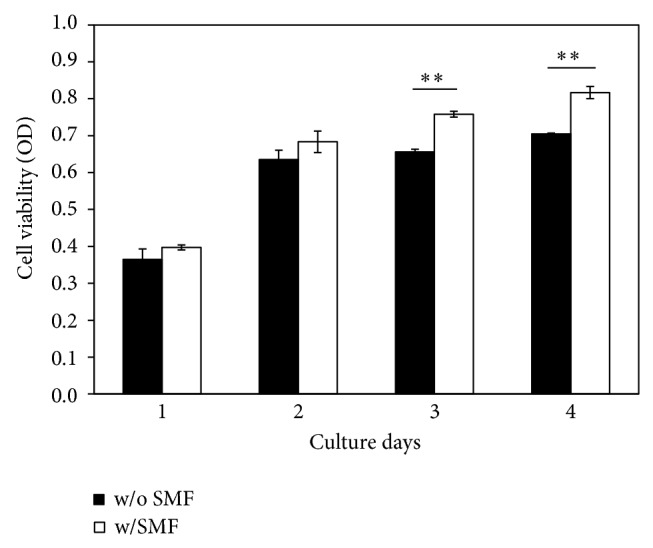
The effect of SMF on the LPS-induced cell viability changes to DPCs. Pretreatment with a 0.4 T SMF significantly attenuated the inflammatory response of LPS.

The MTT assay showed significantly higher cell viability (*P* < 0.001) for SMF-exposed DPCs compared to sham-exposed cells (Figure 6). Cell viability increased up to 15% in SMF-exposed groups during day 3 and day 4. That is, DPCs exposed to a 0.4 T SMF demonstrated a higher proliferation rate compared with the sham-exposed DPCs (Figure 7). Cell viability of DPCs incubated with serial diluted LPS concentrations for 12 hours was also measured by MTT assay. When DPCs were incubated with LPS with concentrations of 400 *μ*g/mL and 600 *μ*g/mL, the tested optical densities decreased to 96.66% and 68.32% of the control values, respectively (Figure 7). However, the results showed that cell viability of LPS-challenged DPCs had significantly higher OD value when cotreated with the 0.4 T SMF (*P* < 0.001). When treated with 400 *μ*g/mL and 600 *μ*g/mL LPS, the optical densities of SMF treated groups were 1.25 and 1.34 times higher than sham-exposed DPCs.

**Figure 8:**
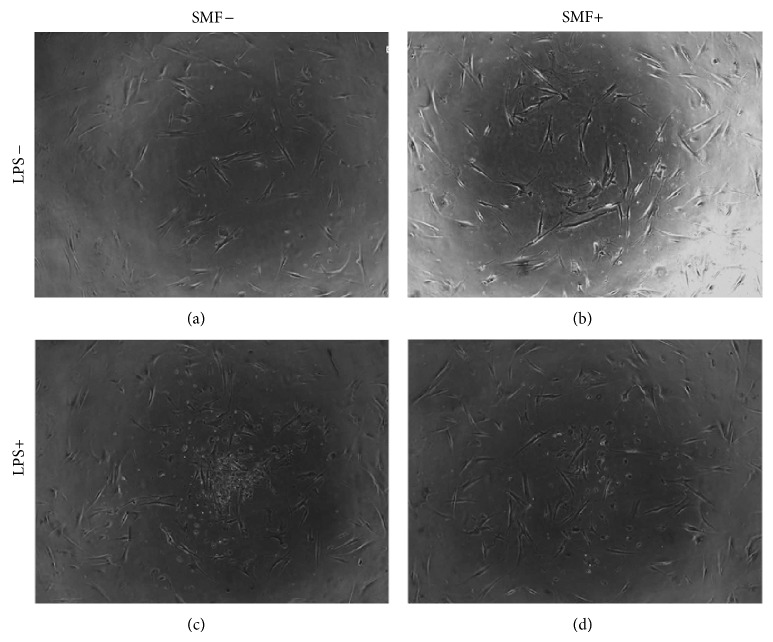
Representation of the optical microscope images of DPC cultures. (a) Sham-exposed DPCs showed fusiform to polygonal shape in a monolayer distribution. (b) There was no difference in cell shape or growth pattern in DPCs exposed to 0.4 T SMF. (c) When challenged with LPS, cell shape changed from fusiform to round, and much suspended debris appeared. (d) However, LPS-challenged DPCs cotreated with 0.4 T SMF had a lower level of shape disorder and suspended debris.

The morphological changes in each experimental group are presented in Figure 8. Sham-exposed DPCs were evenly distributed and formed a continuous monolayer throughout each well (Figure 8(a)). The SMF-exposed DPCs had no obvious changes when compared with control group (Figure 8(b)). Otherwise, more-rounded shape in cell form and suspended debris was observed in the LPS-challenged group (Figure 8(c)). Interestingly, LPS treatment caused less cell pattern change and debris emergence when cells were cotreated with 0.4 T SMF (Figure 8(d)).

**Figure 9:**
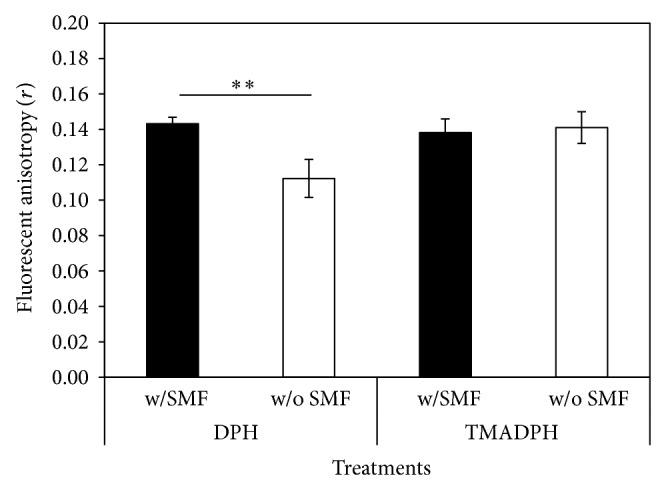
Comparison of fluorescent anisotropy change in DPCs exposed to 0.4 T SMF. Higher fluorescent anisotropy of SMF-exposed cells was found when labeled with DPH.

There was no significant difference in fluorescent anisotropy between SMF-exposed and sham-exposed cells when labeled with TMA-DPH (Figure 9). However, the average DPH fluorescent anisotropy of the exposed cells (0.14) was significantly higher (*P* < 0.001) than the sham-exposed group (0.11). The higher fluorescent anisotropy represents the limited orientation of intercalated DPH. This result suggests that 0.4 T SMF increased the order of hydrophilic region of cell membrane and enhanced the rigidity of lipid bilayer.

## 4. Discussion

It was reported that approximately 1% of pulp cells have the potential to differentiate into odontoblast-like cells and secrete proteins for forming dentin [24]. In this study, immunostaining of various surface markers was performed by flow cytometry. The identity of the DPSCs was confirmed by negative expression of hematopoietic markers CD14 and CD34 and positive expression of CD29, CD73, CD 105, and CD146. In addition, the high expression of CD29, CD73, CD90, and CD105 coincides with other studies [22, 25]. Thus, the DPC sample contain cells meeting criteria of DPSCs.

In 2010, Hsu and Chang reported that the response in proliferation rates of rat dental pulp cells to SMF is insensitivity [26]. They exposed these cells to a 290 mT SMF and found no visible change in cell proliferation rates. However, their results showed that SMF can be an adjuvant to accelerate the osteogenic differentiation and mineralization of cells when rat dental pulp cells were cultured with an osteogenic induction medium combined with SMF exposure [27]. In this study, we found that continuous exposure to a 0.4 T SMF does not affect the multidifferentiation capability of stem cells (Figures 3–5). However, an increase in osteogenic differentiation was not observed in this study. This is because the SMFs were not provided during the osteogenic induction process. Again, enhancement of the proliferation of SMF-exposed human DPCs was found in this study; viability of SMF-exposed cells was 15% higher when compared with sham-exposed cells after 3 days of culturing.

The aim of this study was to investigate whether or not SMFs reduced the toxicity effect of LPS when added to DPCs. LPS was found in apical tissues as well as root canals during endodontic infection [27, 28]. Previous studies reported that this molecule is potentially harmful to host cells as a toxin and as an immune stimulant [5, 28]. Our data also shows that LPS has a toxic effect on DPCs in a dose-dependent manner (Figure 7). Interestingly, however, cell viability of the LPS-challenged DPCs exposed to a 0.4 T SMF was 25% higher than that of the sham-exposed group (Figure 7). These results can be compared with the microscopic observations (Figure 8), which show that development of endotoxin tolerance in the dental pulp cells occurred after 12 hours of continuous 0.4 T SMF exposure. This effect may result from the reduction of proinflammatory cytokine release and increase of anti-inflammatory cytokine release by fibroblasts.

Several studies suggest that DPCs are involved in immune response during pulpal infection through the activation of IL-1 [5–7, 29]. Lin et al. found that long-term continuous exposure to a static magnetic field reduces lipopolysaccharide-induced inflammatory response of fibroblasts by increasing the production of IL-1 receptor antagonist [18]. It was reported that the plasma membrane is the primary site where SMF effect is seen [30, 31]. This effect can also be found in DPCs. Lin et al. used SMFs to improve DPC membrane stability which resulted in a reduction in damage caused by ice crystals during a freezing procedure [32]. In this study, after exposure to a 0.4 T SMF, the DPC membrane fluorescence anisotropy was significantly higher than in the sham-exposed group (Figure 9). These results are consistent with previous studies which also found that 0.4 T SMF increases cell membrane rigidity of MG-63, microglia cells, and red blood cells [17, 33–35]. Since phospholipids can be oriented by external magnetic fields when they are exposed to flux densities exceeding a certain threshold [36–38], dental pulp cell membranes can be altered and the binding capability between LPS and its cross membrane receptor, Toll-like receptor 4, can also be changed.

Based on these findings, it appears reasonable to suggest that SMF stimulation inhibits LPS-induced inflammatory response of dental pulp cells. Moreover, SMF exposure can also enhance the proliferation of dental pulp cells in the later days. Therefore, although more advanced studies are needed, we suggested that SMF can be a possible choice to be used in clinical practice to treat LPS infected dentine-pulp complex.
